# Clinical pharmacy services for tuberculosis management: a systematic review

**DOI:** 10.3389/fphar.2023.1186905

**Published:** 2023-07-07

**Authors:** D. Iskandar, F. D. A. Suryanegara, J. F. M. van Boven, M. J. Postma

**Affiliations:** ^1^ Unit of Global Health, Department of Health Sciences, University Medical Center Groningen, University of Groningen, Groningen, Netherlands; ^2^ Faculty of Pharmacy, Bhakti Kencana University, Bandung, Indonesia; ^3^ Department of Pharmacy, Faculty of Mathematics and Natural Sciences, Universitas Islam Indonesia, Yogyakarta, Indonesia; ^4^ Department of Clinical Pharmacy and Pharmacology, University Medical Center Groningen, University of Groningen, Groningen, Netherlands; ^5^ Groningen Research Institute for Asthma and COPD (GRIAC), University Medical Center Groningen, University of Groningen, Groningen, Netherlands; ^6^ Centre for Medicine Use and Safety, Monash Institute of Pharmaceutical Sciences, Monash University, Melbourne, VIC, Australia; ^7^ Department of Economics, Econometrics, and Finance, Faculty of Economics and Business, University of Groningen, Groningen, Netherlands; ^8^ Department of Pharmacology and Therapy, Faculty of Medicine, Universitas Airlangga, Surabaya, Indonesia; ^9^ Center of Excellence in Higher Education for Pharmaceutical Care Innovation, Universitas Padjadjaran, Bandung, Indonesia

**Keywords:** clinical pharmacy, pharmaceutical care, pharmacist intervention, treatment outcomes, tuberculosis

## Abstract

**Objective:** This study aims to systematically review the content and potential effects of clinical pharmacy services in tuberculosis (TB) care management.

**Methods:** Searches were performed in PubMed, Embase, Cochrane, Scopus, and Web of Science databases following Preferred Reporting Items for Systematic Reviews and Meta-Analysis (PRISMA) guidelines. Study characteristics and outcomes were extracted, and clinical pharmacy service components were characterized using the Descriptive Elements of Pharmacist Intervention Characterization Tool.

**Results:** Twenty articles were included for full-text assessment, of which 10 fulfilled inclusion criteria, comprising 1,168 patients (*N* = 39 to 258 per study). These articles included five prospective cohort studies, two case–control studies, two quasi-experimental studies, and one cross-sectional study. Intervention foci within clinical pharmacy services were medication adherence (50%), medication safety (40%), education to patients/caregivers regarding needs/beliefs (30%), optimizing medication/therapy effectiveness (30%), emphasizing HRQoL (10%), and drug selections (10%). The three most frequently applied interventions were drug information/patient counseling (80%), adverse drug reaction monitoring (50%), and drug use evaluation (20%). Based on the World Health Organization (WHO) outcome classification, treatment success ranged from 72% to 93%, with higher cure outcomes (53%–86%) than treatment completion (7%–19%). Other outcomes, including isoniazid metabolites, medication counts, sputum conversion, adherence/compliance, knowledge, and quality of life, were better in the intervention group than those in comparator groups, and/or they improved over time. Risk of bias analysis indicated that the included studies were not comparable to a randomized clinical trial.

**Conclusion:** Clinical pharmacy services as single or composite interventions potentially improve TB outcomes, but its evidence is still inconsistent and limited due to the lack of randomized controlled studies using the WHO outcome classification.

**Systematic review registration:** https://www.crd.york.ac.uk/prospero/display_record.php?RecordID=199028, identifier CRD42020199028.

## Introduction

Tuberculosis (TB), as one of the leading causes of death from a single infectious agent, is still a disease of major global concern (World Health Organization, 2014). In 2020, 10 million people developed TB globally, followed by an 18% decline in the number of people newly diagnosed with TB between 2019 and 2020 (World Health Organization, 2021a). Steps have been taken to reduce the number of TB cases and deaths through improvements in the financing, research, innovation, and development of a multi-sectoral accountability framework (World Health Organization, 2019a; World Health Organization, 2019b).

Between 2000 and 2020, TB treatment averted 66 million deaths (World Health Organization, 2021a) as effective and timely TB treatment affects patients’ survival and potentially decreases *M. tuberculosis* transmission indirectly (Schoenbaechler et al., 2021; Tierney et al., 2021). Additionally, supervised treatment increases the treatment success probability of new TB cases (Wen et al., 2018), avoiding retreatment, which has a similar failure likelihood when the initial treatment fails (Cohen et al., 2018).

In 2019, there was an 86% treatment success rate for people treated for TB with first-line regimens, accompanied by increasing rifampicin resistance, from 61% in 2019 to 71% in 2020 (World Health Organization, 2021a). Hence, initial treatment warrants particular concerns because failing increases resistance probability (van der Werf et al., 2012; DiPiro et al., 2017) and affects treatment outcomes (Chaves Torres et al., 2019). When resistance develops, the drug of choice being based on a susceptibility test is a significant treatment outcome predictor (Gegia et al., 2017; Ahmad et al., 2018) because using more potent or newer drugs or fixed-dose combinations (FDCs) does not guarantee favorable outcomes in the potential absence of adequate susceptibility (Albanna et al., 2013; Lee et al., 2016; Xu et al., 2017). In the case of resistance, drug effectiveness and adverse drug events are of major concern; for that matter, appropriate monitoring by involving a pharmacist could better optimize the therapy and adverse event management (Khan et al., 2022).

In general, pharmacist services positively affect patient outcomes (Shrestha et al., 2022), especially when targeting specific conditions using well-defined interventions and outcome measures (Rotta et al., 2015); therefore, with the current significant TB burden, pharmacists’ active involvement in treating and managing TB cases could be a valuable resource for further improving pharmacological treatment outcomes.

Pharmaceutical, patient-centered care delivered as clinical pharmacy services (CPS) by pharmacists aims to support patients in achieving optimal drug outcomes within a multi-disciplinary healthcare team (Franklin and van Mil, 2005; Whittlesea and Hodson, 2019; Negaard et al., 2020). Conceptually, pharmaceutical care and CPS are often overlapping. Pharmaceutical care is about cooperative systems and relationship ethics, not pharmacists or technical functions *per se*, which drive CPS activities (Dreischulte and Fernandez-Llimos, 2016). Pharmaceutical care is an extension of CPS (Hepler, 2004), while CPS itself is more of a scientific discipline of rational medication use, empowering pharmacists to provide patient-centered interventions (Hepler, 2004; American College of Clinical Pharmacy, 2008; Holdford and Brown, 2010; Dreischulte and Fernandez-Llimos, 2016). CPS can be seen as a professional practice of pharmacists, which is a building block of pharmaceutical care that requires adequate knowledge and skill in pharmacists for delivering the intervention or providing treatment. Having a clear picture of the contribution of clinical pharmacy services to broader pharmaceutical care services in TB care is essential in improving TB care further. Hence, this study aims to systematically review the content and potential effects of clinical pharmacy services in TB care management.

## Methods

### Study design

A systematic review was performed and reported according to the “Preferred Reporting Items for Systematic Reviews and Meta-Analysis” (PRISMA) statement (Page et al., 2021). The study has been registered at the International Prospective Register of Systematic Reviews (PROSPERO) with the number CRD42020199028.

### Eligibility criteria

Peer-reviewed publications meeting the following inclusion and exclusion criteria were eligible:

Inclusion criteria: published studies in English, either observational or intervention studies of clinical pharmacy services in TB care management in the hospital setting in adult patients (≥18 years) with confirmed drug-susceptible TB or (extended or multi) drug-resistant TB by microbiological verification, were included.

Exclusion criteria: we excluded non-full-text studies, conference reports, reviews, editorials, comments, or letters. Studies focused on TB patients with specific comorbidities, like diabetes or HIV, were also excluded.

It should be noted that post-protocol registration in the PROSPERO, we decided to restrict our inclusion to drug-susceptible TB studies only, excluding drug-resistant TB studies. This was carried out to reduce heterogeneity and enhance result interpretation, given that the initially included studies encompassed a very broad variety of interventions and outcomes.

### Information sources

The systematic literature search was performed on 20 September 2021, in PubMed, Embase, Cochrane, Scopus, and Web of Science databases.

### Search strategy

The search was carried out using Medical Subject Headings (MeSHs) and other relevant subject headings and text words. The main categories were “tuberculosis” and “pharmacy service,” which were combined by the Boolean operator “AND.” The “OR” operator combined each category’s subject headings and text words. A detailed overview of the search strategy is provided (see Supplementary Appendix, Search Strategy).

### Selection process

The article screening process was performed using Rayyan (https://rayyan.ai/). Rayyan is an electronic tool that provides consistent exclusion reasoning corresponding to the study criteria of language, publication type, study design, intervention, subject, medication, and outcomes. Specific requirements were established from each study criteria, and articles not fulfilling these criteria were excluded with an assigned exclusion reason label (see Supplementary Appendix Table S1). To assist the screening process, article screening criteria were complemented with an article screening flowchart, according to the Rayyan workflow (see Supplementary Appendix Figure S1). In addition, those articles’ important text terms and metadata were analyzed using VOSviewer (https://www.vosviewer.com/) to describe and visualize the bibliometric network.

Two authors (DI and FDA) independently screened the titles and abstracts of all studies identified by the search strategy based on inclusion and exclusion criteria, guided by the article screening criteria and flowchart. First, duplicated articles across databases were removed; then, the title and abstract of the articles were screened to find relevant and potential articles for further full-text reviews. Any disagreement was resolved through discussion until consensus was achieved.

### Data collection process

Two authors (DI and FDA) used an electronically pre-designed form created using REDCap (https://redcap.umcg.nl/redcap/) to collect and extract data from each included study independently. The extraction forms captured information related to the study characteristics, clinical pharmacy services, outcomes, and risk of bias, as further specified in Data items.

### Data items

The following data were extracted: 1) study characteristics, including first author and year of study publication, study design, duration, setting, number of participants, and location; 2) clinical pharmacy service components, consisting of clinical data sources, interventions (focus and activities), and supporting materials; 3) outcomes, including TB care management treatment outcomes as per the World Health Organization (WHO) classifications (cured, treatment completed, treatment failed, defaulted, and transfer) (World Health Organization, 2010) and other outcomes that include process outcomes, such as patient satisfaction and medication adherence; and 4) risk of bias, including the bias due to confounding, participant selection, intervention classification, intervention deviation, missing data, outcomes, and reporting. The latter is further described in the “Risk of bias assessment”.

A taxonomy tool was used to identify and classify the study design when the included studies were not clearly stating it (see Supplementary Appendix Table S2). For extracting clinical pharmacy service information, the Descriptive Elements of Pharmacist Intervention Characterization Tool (DEPICT) was used (see Supplementary Appendix Table S3). The DEPICT provides consistent and structured information, avoiding variability in the naming or nomenclature of common pharmacy service conceptual frameworks and constructs (Correr et al., 2013).

Intervention focus refers to the parameters pharmacists evaluated, while intervention activities are actions carried out by pharmacists to address the identified problem.

### Risk of bias assessment

When there was no limitation to the study design, all retrieved studies included in the review were non-randomized (see Result). The Risk of Bias in Non-randomized Studies—of Intervention (ROBINS-I) was used to appraise the potential bias in studies without randomization (Sterne et al., 2016). The main feature of this assessment is to compare whether the study systematically differs from a well-designed and performed randomized trial. The bias is low when the study is comparable, moderate when it has sound evidence but is not comparable, serious when it has problematic aspects of its design and performance, and critical when it is unlikely to provide valuable evidence. The following dimensions were assessed individually before concluding about its overall bias: bias due to confounding, selection of participants, classification of the intervention, deviation from the intended intervention, missing data, measurements of outcomes, and selection of the reported result. Two reviewers independently assessed the articles based on the table and guidelines provided by the development group for ROBINS-I (Development Group for ROBINS-I, 2016). A joint review and consensus between reviewers resolved any disagreement. The result was visualized using R statistics version 4.1.3 (https://www.r-project.org/) based on the robvis package (McGuinness and Higgins, 2020).

## Results

### Study selection

**FIGURE 1:**
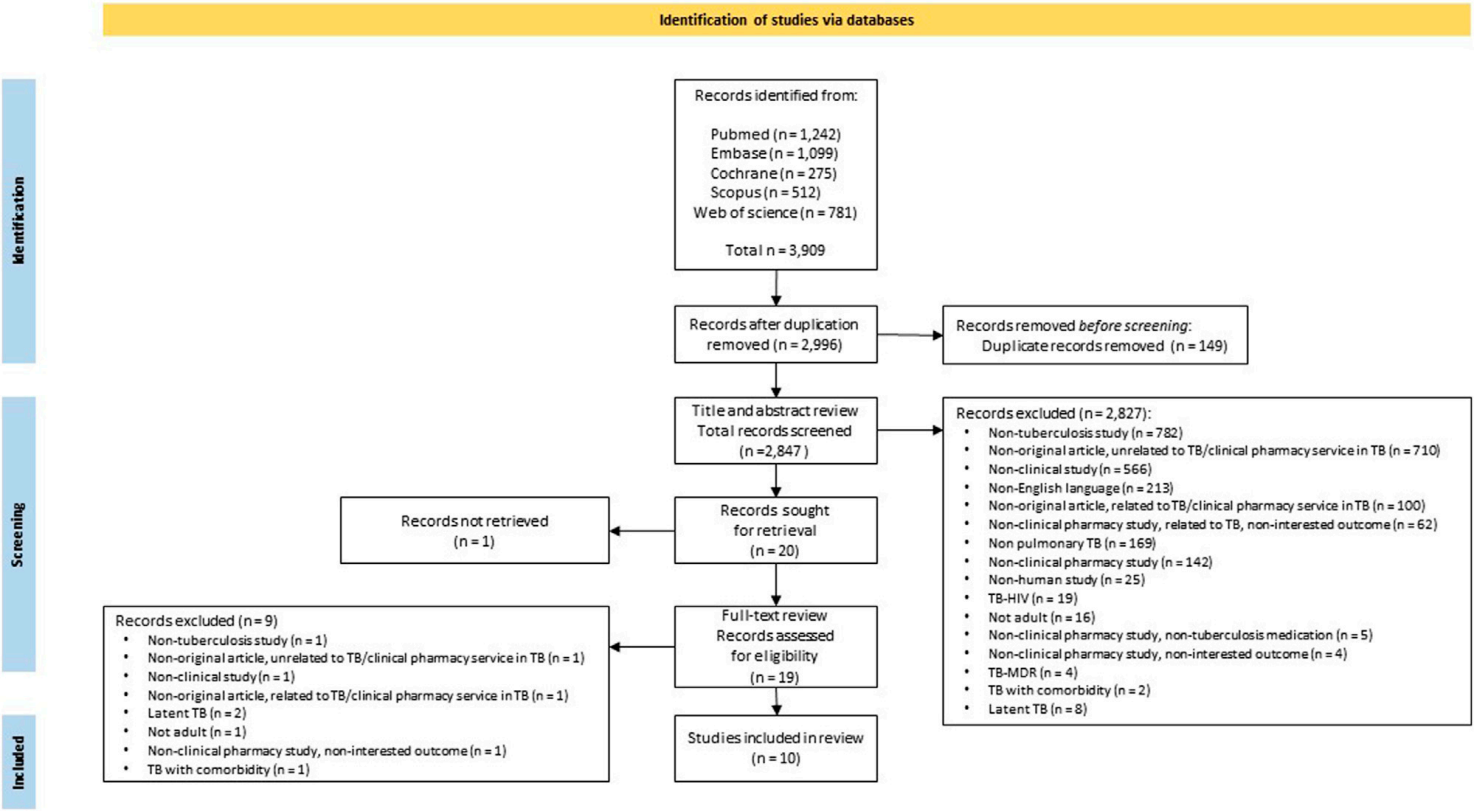
PRISMA flow diagram of the study identification process.

The study selection flow is presented in Figure 1. In total, after applying the search strategy, 3,909 publications were retrieved. For the 2,996 articles obtained after duplicate removal, a bibliometric network analysis based on important text terms was carried out, resulting in five clusters of articles. One cluster related to the keywords of pharmacist, health, and service was identified. This cluster represents pharmacist health services in TB care management (see Supplementary Appendix Figure S2). Subsequently, 2,847 articles were screened after further manual checking and removing duplicates. No unique focus on TB was the main exclusion reason. Other predominant reasons for exclusion were non-original articles, no clinical pharmacy services, and no clinical study.

Twenty articles were included for full-text retrieval upon the consensus of the two authors. Finally, 10 articles were included in the review based on the eligibility assessment and consensus between the two authors.

Table 1 provides an overview of the included studies. Six studies originated from high-TB-burden countries, including four from India (Bhardwaja et al., 2012; Venkatapraveen et al., 2012; Thomas et al., 2018; Narayana et al., 2020), one from China (Tang et al., 2018), and one from Indonesia (Karuniawati et al., 2019). Others were from different regions: Thailand (Tanvejsilp et al., 2018), Ivory Coast (Abrogoua et al., 2016), Türkiye (Clark et al., 2007), and Brazil (Lopes et al., 2017). The earliest included study was published in 2007. Notably, among the 10 studies, no randomized controlled studies were identified. Study designs were heterogeneous and included five prospective cohort studies (50%) (Bhardwaja et al., 2012; Venkatapraveen et al., 2012; Lopes et al., 2017; Tanvejsilp et al., 2018; Thomas et al., 2018), two case–control studies (20%) (Clark et al., 2007; Tang et al., 2018), two quasi-experimental studies (with and without control) (Karuniawati et al., 2019; Narayana et al., 2020), and a cross-sectional study (Abrogoua et al., 2016). Most studies were prospective; for only one study, this was unknown (Abrogoua et al., 2016). Most study settings were in the tertiary care hospital, except for one, which was in a secondary care hospital (Narayana et al., 2020). The study duration ranged from 5 to 35 months, and patient numbers varied between 39 and 258.

**TABLE 1:** Characteristics of the included studies on clinical pharmacy services for TB care management.

Author, year	Country	Study design	Setting	Number of participants	Population	Intervention	Follow-up	Outcome
Abrogoua et al. (2016)	Ivory Coast	Cross-sectional study	Tertiary care hospital	130	All TB inpatients	Drug regimen review	Not applicable	Pharmaceutical intervention acceptance rate
Bhardwaja et al. (2012)	India	Prospective cohort study	Tertiary care hospital	39	Patients diagnosed and undergoing tuberculosis treatment	Adherence assessment and counseling	Baseline, first month, and second month	Morisky Medication Adherence Scale
Clark et al. (2007)	Turkey	Prospective case–control study	Tertiary care hospital	114	Patients newly diagnosed with TB and receiving first-line anti-TB drugs	Education by the clinical pharmacist shortly before discharge (as well as the routine medical and nursing care) and pharmaceutical care needs/issues assessment	Post discharge, first month, second month, and fourth month	Attendance, isoniazid metabolites, medication counting, and resolution of pharmaceutical care needs/issues
				40	Patients recently diagnosed with MDR-TB and receiving second-line anti-TB treatment	Pharmaceutical care needs/issues assessment	During initial treatment (inpatient) and bi-monthly during outpatient (until study completion)	Resolution of pharmaceutical care needs/issues
Karuniawati et al. (2019)	Indonesia	Quasi-experiment (with control)	Tertiary care hospital	75	Outpatients with pulmonary tuberculosis	Counseling and counseling with leaflets	Pre- and post-intervention	Compliance in taking medication and adherence level
Lopes et al. (2017)	Brazil	Prospective cohort study	Tertiary care hospital	62	Patients diagnosed with TB and receiving pharmaceutical care	Adverse drug reaction detection;	Unspecified	Pharmaceutical care impact index
						Non-pharmacological (guidance on the proper use of drugs and their risks and referral to other health professionals);		
						Pharmacological (suspension of, addition of, or change in medication)		
Narayana et al. (2020)	India	Quasi-experiment (without control)	Secondary care hospital	258	Patients diagnosed with smear-positive pulmonary tuberculosis under isoniazid (INH), rifampicin (RIF), and pyrazinamide (PZA) in their treatment regimen	Education/counseling	Baseline, third month, and sixth month	Knowledge level and adherence level
Tang et al. (2018)	China	Prospective case–control study	Tertiary care hospital	131	Newly diagnosed patients with pulmonary tuberculosis who had received their first anti-tuberculosis drugs for <1 month	Pharmaceutical care: patient education, urine analysis for the presence of isoniazid metabolites, medication counting, monitoring laboratory tests, and addressing patient-reported pharmaceutical care issues	First month, second month, and sixth month	Treatment success (cure or treatment completion), failure, transfer out, default, death, attendance, isoniazid metabolites, and medication counting
Tanvejsilp et al. (2018)	Thailand	Prospective cohort study	Secondary and tertiary care hospitals	104	Patients with confirmed pulmonary TB by a physician and being classified bacteriologically as smear positive or smear negative	Pharmaceutical care (pharmacist-led patient education at every outpatient visit), home visit (regular home visit until treatment completion), and SAT (patients take medications by themselves at home without any additional supportive approach)	Initial (within 2 months of the intensive phase), fourth month, and sixth month	Quality of life
Thomas et al. (2018)	India	Prospective cohort study	Tertiary care hospital	95	Patients diagnosed and undergoing TB treatment	Education/counseling	Second month, fourth month, and sixth month	Morisky Medication Adherence Scale and adherence barrier
Venkatapraveen et al. (2012)	India	Prospective cohort study	Tertiary care hospital	120	Pulmonary tuberculosis patients	Education/counseling	Baseline, sixth week, and 12th week	Knowledge and adherence score, and sputum conversion

SAT: self-administered therapy.

### Clinical pharmacy services

The clinical pharmacy service component comprised clinical data sources, intervention focus, intervention activities, and supporting materials identified using the DEPICT.

Of the 10 studies included in the review, clinical data sources used for clinical pharmacy services were varied; seven studies used adherence measuring tools (70%) (Clark et al., 2007; Bhardwaja et al., 2012; Venkatapraveen et al., 2012; Tang et al., 2018; Thomas et al., 2018; Karuniawati et al., 2019; Narayana et al., 2020), six used medical records (60%) (Clark et al., 2007; Bhardwaja et al., 2012; Abrogoua et al., 2016; Tang et al., 2018; Tanvejsilp et al., 2018; Thomas et al., 2018), five used patient interviews (50%) (Clark et al., 2007; Bhardwaja et al., 2012; Lopes et al., 2017; Tanvejsilp et al., 2018; Thomas et al., 2018), and four used laboratory testing/therapeutic drug monitoring (40%) (Clark et al., 2007; Venkatapraveen et al., 2012; Tang et al., 2018; Thomas et al., 2018), amongst others. The intervention focus provided within the clinical pharmacy services was medication adherence, defined as the voluntary cooperation of the patient in taking the drugs or medicine as prescribed (DEPICT, 2013), in five studies (50%) (Clark et al., 2007; Bhardwaja et al., 2012; Tang et al., 2018; Thomas et al., 2018; Karuniawati et al., 2019); medication safety in four studies (40%) (Clark et al., 2007; Abrogoua et al., 2016; Lopes et al., 2017; Tang et al., 2018); education of patients/caregivers regarding the needs/beliefs in three studies (30%) (Venkatapraveen et al., 2012; Thomas et al., 2018; Narayana et al., 2020); optimizing the medication/therapy effectiveness in three studies (30%) (Venkatapraveen et al., 2012; Tang et al., 2018; Tanvejsilp et al., 2018); and lastly, emphasis on HRQoL (10%) (Tanvejsilp et al., 2018) and drug selections (10%) (Abrogoua et al., 2016) in one study (see Supplementary Appendix Figures S3, S4).

In all studies, intervention activities that corresponded to intervention focus within clinical pharmacy services for TB management could include single (Bhardwaja et al., 2012; Venkatapraveen et al., 2012; Lopes et al., 2017; Thomas et al., 2018; Karuniawati et al., 2019; Narayana et al., 2020) or composite (Clark et al., 2007; Abrogoua et al., 2016; Tang et al., 2018; Tanvejsilp et al., 2018) activities (see Supplementary Appendix Table S4).

**FIGURE 2:**
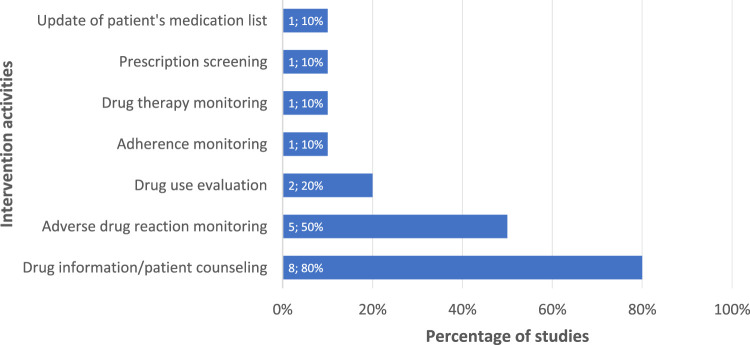
Intervention activities in clinical pharmacy services for TB care management.

The three most frequently applied intervention activities identified from 10 included studies were drug information/patient counseling (Clark et al., 2007; Bhardwaja et al., 2012; Venkatapraveen et al., 2012; Tang et al., 2018; Tanvejsilp et al., 2018; Thomas et al., 2018; Karuniawati et al., 2019; Narayana et al., 2020), adverse drug reaction monitoring (Clark et al., 2007; Abrogoua et al., 2016; Lopes et al., 2017; Tang et al., 2018; Tanvejsilp et al., 2018), and drug use evaluation (Abrogoua et al., 2016; Tang et al., 2018), as presented in Figure 2. Further details on the type of intervention activities based on their corresponding intervention focus are shown in the Supplementary Appendix Table S5. Aligned with intervention activities, the mainly used supporting materials for interventions were educational materials/leaflets/written action plans, used in seven studies (70%) (Clark et al., 2007; Bhardwaja et al., 2012; Venkatapraveen et al., 2012; Tang et al., 2018; Thomas et al., 2018; Karuniawati et al., 2019; Narayana et al., 2020); patient data collection forms in four studies (40%) (Bhardwaja et al., 2012; Venkatapraveen et al., 2012; Tanvejsilp et al., 2018; Thomas et al., 2018); and validated adherence questionnaires in three studies (30%) (Venkatapraveen et al., 2012; Karuniawati et al., 2019; Narayana et al., 2020), amongst others (see Supplementary Appendix Figure S5).

### Outcomes

The outcomes of clinical pharmacy services for TB care management varied as per the study. An overview is provided in Table 2.

**TABLE 2:** Outcomes of clinical pharmacy services for TB care management by intervention focus.

Intervention focus	Outcome
Drug selection	Pharmaceutical intervention acceptance rate (Abrogoua et al., 2016)
HRQoL	EQ-5D-3L score (Tanvejsilp et al., 2018)
	VAS (Tanvejsilp et al., 2018)
Medication/therapy effectiveness	Cure (Tang et al., 2018; Tanvejsilp et al., 2018)
	Death (Tang et al., 2018; Tanvejsilp et al., 2018)
	Default (Tang et al., 2018; Tanvejsilp et al., 2018)
	Failure (Tang et al., 2018)
	Not completed (Tanvejsilp et al., 2018)
	Transfer out (Tang et al., 2018)
	Treatment completion (Tang et al., 2018; Tanvejsilp et al., 2018)
	Treatment success (Tang et al., 2018)
	Sputum conversion (%) (Venkatapraveen et al., 2012)
	Sputum conversion time (months) (Tang et al., 2018)
Medication adherence	Adherence level (Karuniawati et al., 2019)
	Attendance (Clark et al., 2007; Tang et al., 2018)
	Compliance (Karuniawati et al., 2019)
	Isoniazid metabolites (Clark et al., 2007; Tang et al., 2018)
	Medication counting (Clark et al., 2007; Tang et al., 2018)
	Morisky Adherence Questionnaire Scale (Bhardwaja et al., 2012; Thomas et al., 2018)
Medication safety	Pharmaceutical care impact index (Lopes et al., 2017)
	Pharmaceutical care needs (Clark et al., 2007; Tang et al., 2018)
	Pharmaceutical intervention acceptance rate (Abrogoua et al., 2016)
Patient/caregiver educational needs/beliefs	Adherence barrier: knowledge (Thomas et al., 2018)
	Adherence barrier: personal (Thomas et al., 2018)
	Adherence barrier: psychological (Thomas et al., 2018)
	Knowledge (Thomas et al., 2018; Narayana et al., 2020)
	Knowledge and adherence score (Venkatapraveen et al., 2012)
	Medication adherence (Narayana et al., 2020)

HRQoL, health-related quality of life; EQ-5D-3L, Euro Quality of Life 5 Dimensions 3 Levels; VAS, visual analog scale.

We classified identified outcomes of interest as WHO outcomes and other outcomes. WHO outcomes are standardized treatment outcomes of TB patients recorded at the treatment course’s end. It includes cure, treatment completion, default, death, treatment success, not completed, transfer out, and failure (World Health Organization, 2010; World Health Organization, 2021b). The rest were classified as other outcomes. Of all the outcomes measured, we reported timings, follow-up, and group comparisons, if reported in the studies.

### WHO outcomes

Two studies (Tang et al., 2018; Tanvejsilp et al., 2018) reported intervention outcomes between different groups as per the WHO classification at the end of the intensive (2 months) and continuation (4 months) phase of TB care, although there were slight differences in the measured outcomes. Percentage values represent the proportion of patients within the group having an outcome as per the WHO classification (Table 3). One study compared a pharmaceutical care group with two different intervention groups (Tanvejsilp et al., 2018), and others compared it to one group, usual care (Tang et al., 2018). The value of “Treatment success” in one study (Tanvejsilp et al., 2018) was calculated from the sum of “Treatment completion” and “Cure” outcomes since it was not readily available.

**TABLE 3:** WHO outcomes of clinical pharmacy services for TB care management.

Outcome	Tang et al. (2018)			Tanvejsilp et al. (2018)			
	Pharmaceutical care	Usual care	*p*-value	Pharmaceutical care	Home visit	SAT	*p*-value
Treatment success	72%	54%	0.137	93.1%^*^	97.3%^*^	83.7%^*^	-
Treatment completion	19%	14%	0.762	6.9%	36.8%	40.5%	0.001
Cure	53%	40%	0.373	86.2%	60.5%	43.2%	
Default	5%	19%	0.052	0%	0%	10.8%	
Death	3%	3%	0.979	0%	2.6%	5.4%	
Not completed	-	-	-	6.9%	0%	0%	
Failure	10%	6%	0.611	-	-	-	-
Transfer out	10%	18%	0.443	-	-	-	-

^a^ Sum of treatment completion and cure outcomes

SAT, self-administered therapy

Treatment outcomes from the study by Tang et al. (2018) for the pharmaceutical care group showed an improvement compared to usual care, except for “Failure” outcomes. However, no statistical significance was found in all the outcomes measured between groups. On the contrary, a comparison between groups in the study by Tanvejsilp et al. (2018) showed that outcome differences were statistically significant when considered as a composite of outcomes, whereas the pharmaceutical care group was not always providing better outcomes.

In Tang et al. (2018), the “Treatment success” in the pharmaceutical care group was higher than that in another group, which was usual care; furthermore, in the study by Tanvejsilp et al. (2018), it was higher than that in the SAT group and lower than the home visit group.

The “Treatment completion” in the pharmaceutical care group from Tang et al. (2018) was higher than that in the usual care group care, yet the study by Tanvejsilp et al. (2018) showed that the pharmaceutical care group had lower values than the home visit and SAT group. For the “Cure” outcome, studies consistently showed that the pharmaceutical care group scored higher than other groups.

For “Default” and “Death” outcomes, the pharmaceutical care group scored lower across studies or at least the same as the other groups (Tang et al., 2018). However, the “Not completed” outcome was higher in the pharmaceutical care group than in the home visit and SAT group (Tanvejsilp et al., 2018). Lastly, the “Failure” outcome in the pharmaceutical care group was higher than the usual care group, and the “Transfer out” outcome was lower (Tang et al., 2018).

### Other outcomes

Unlike WHO outcomes, other outcomes were primarily process outcomes related to the intervention given by the pharmacist and were reported at the end or at a specific follow-up time interval within the intensive and continuation phase. Table 4 presents outcomes measured at the end of the intensive and continuation phase identified in four studies (Clark et al., 2007; Venkatapraveen et al., 2012; Tang et al., 2018; Karuniawati et al., 2019), and Table 5 shows outcomes measured at a specific follow-up time interval within the intensive and continuation phase in three studies (Venkatapraveen et al., 2012; Tanvejsilp et al., 2018; Karuniawati et al., 2019). One study measured both outcomes at the end or at a specific follow-up time interval within intensive and continuation phases (Venkatapraveen et al., 2012).

**TABLE 4:** Other outcomes of clinical pharmacy services for TB care management measured at the end of the intensive and continuation phases.

Outcome	Clark et al. (2007)				Tang et al. (2018)		Venkatapraveen et al. (2012)		Karuniawati et al. (2019)			
	No education	Education	*p*-value	Usual care	Pharmaceutical care	*p*-value	No education	Education	No counseling and leaflet	Counseling	Counseling and leaflet	*p*-value
Attendance	29.30%	53.60%	<0.01	61.00%	81.00%	0.018	-	-	-	-	-	-
Isoniazid metabolites	42.30%	80.40%	<0.001	50.00%	80.00%	0.002	-	-	-	-	-	-
Medication counting	85.8% ± 9.4%	88.7% ± 10.7%	-	84.80%	89.10%	0.264	-	-	-	-	-	-
Sputum conversion time (months)	-	-	-	2.97 ± 2.21	2.78 ± 2.06	0.708	-	-	-	-	-	-
Sputum conversion (percentage)	-	-	-	-	-	-	43.86%	80.71%	-	-	-	-
Adherence level	-	-	-	-	-	-	-	-	12	13	13	<0.001

For “Attendance” and “Isoniazid metabolites” outcomes, the intervention group [education (Clark et al., 2007) or pharmaceutical care (Venkatapraveen et al., 2012)] provided higher values and significantly differed from the no education (Clark et al., 2007) or usual care group (Tang et al., 2018). However, it was not statistically significant for “Medication counting” (Clark et al., 2007; Tang et al., 2018). “Sputum conversion” also improved, although its significance was inconclusive (Venkatapraveen et al., 2012). The “Adherence level” was improved and was statistically significant (Karuniawati et al., 2019).

**TABLE 5:** Other outcomes of clinical pharmacy services for TB care management measured at a follow-up time interval within the intensive and continuation phases.

Outcome	Venkatapraveen et al. (2012)		Karuniawati et al. (2019)			Tanvejsilp et al. (2018)		
	No education	Education	No counseling and leaflet	Counseling	Counseling and leaflet	SAT	Home visit	Pharmaceutical care
Knowledge and adherence score								
Baseline	6.8	6.7	-	-	-	-	-	-
First follow-up	7.93	10.72	-	-	-	-	-	-
Second follow-up	8.84	12.27	-	-	-	-	-	-
*p*-value	<0.0001	<0.0001	-	-	-	-	-	-
Compliance in taking medication								
Pre	-	-	40.00%	36.00%	44.00%	-	-	-
Post	-	-	24.00%	56.00%	84.00%	-	-	-
*p*-value	-	-	0.537	0.029	0.003	-	-	-
EQ-5D-3L score								
Start	-	-	-	-	-	0.738 ± 0.178	0.72 ± 0.21	0.661 ± 0.206
End	-	-	-	-	-	0.913 ± 0.13	0.905 ± 0.149	0.83 ± 0.269
*p*-value	-	-	-	-	-	<0.001	<0.001	0.021
VAS								
Start	-	-	-	-	-	66.43±	61.5% ± 24.06%	68.46% ± 15.15%
End	-	-	-	-	-	94% ± 7.51%	91.94% ± 8.39%	89.92% ± 9.62%
*p*-value	-	-	-	-	-	<0.001	<0.001	<0.001

SAT, self-administered therapy; EQ-5D-3L, Euro Quality of Life 5 Dimensions 3 Levels; VAS, visual analog scale

Percentage values of attendance outcomes referred to the proportion of patients who adhered to a predetermined visit/monitoring schedule. For isoniazid metabolites, this included the proportion of patients with detected isoniazid metabolites in their urine samples. For medication counting, this was the proportion of medications the patient took. Sputum conversion identifies the result of a sputum test from positive to negative for *M. tuberculosis*, reported as a time interval or as the proportion of patients having a negative result.

Of the three studies included, the outcomes were specific for each study. For “Knowledge and adherence score” and “Compliance” outcomes, interventions of Education (Venkatapraveen et al., 2012) and counseling and leaflet (Karuniawati et al., 2019) showed statistically significant improvements over time. There was a significant improvement in the quality of life measures within the pharmaceutical care group; however, it was lower than the SAT and home visit group (Tanvejsilp et al., 2018). The percentage of compliance in taking medicines exhibited a proportion of patients who adhere to the medication schedule.

### Risk of bias assessment

**FIGURE 3:**
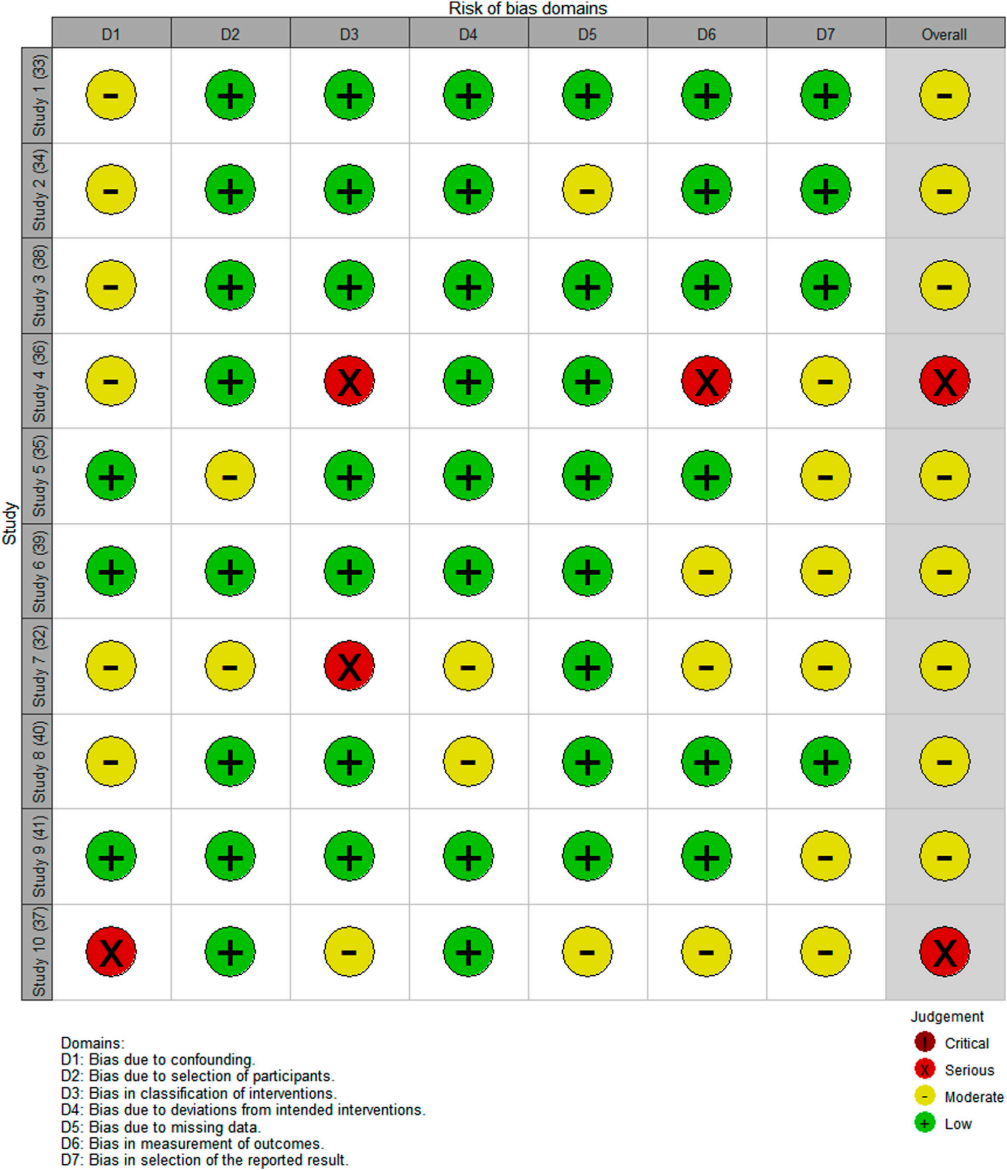
Overall risk of bias.

**FIGURE 4:**
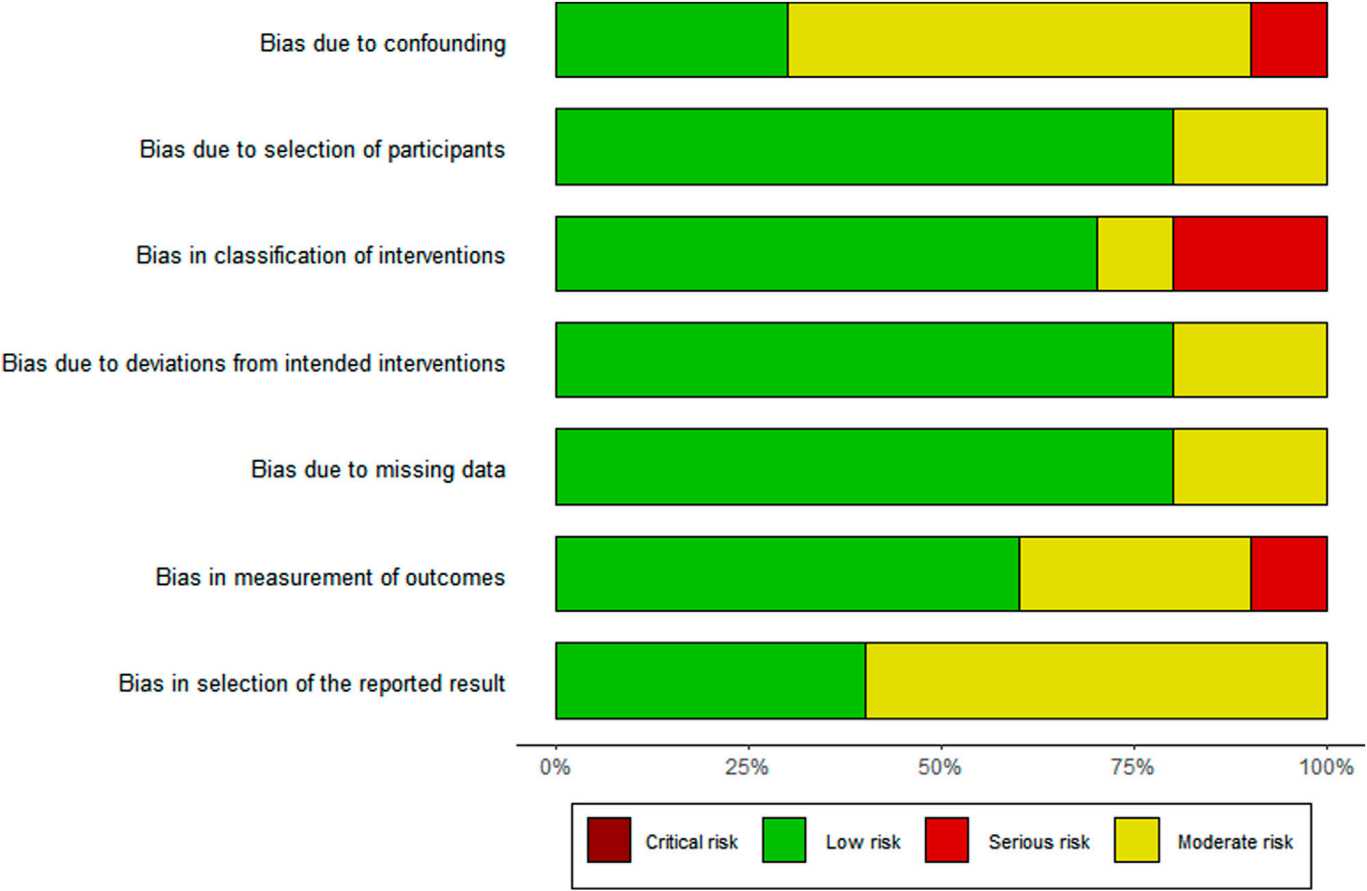
Risk of bias per domain.

The risk of bias assessment is visually presented overall per study and per domain in Figures 3, 4.

Of the 10 studies assessed, the overall bias for eight studies was moderate (Clark et al., 2007; Bhardwaja et al., 2012; Venkatapraveen et al., 2012; Abrogoua et al., 2016; Tang et al., 2018; Tanvejsilp et al., 2018; Thomas et al., 2018; Karuniawati et al., 2019) and in two, it was serious (Lopes et al., 2017; Narayana et al., 2020). Commonly identified factors contributing to moderate bias occurrence were no group comparisons in the study (Bhardwaja et al., 2012; Venkatapraveen et al., 2012; Abrogoua et al., 2016; Lopes et al., 2017; Thomas et al., 2018; Narayana et al., 2020), patient selection (Tanvejsilp et al., 2018), intervention on the baseline patient assessment (Clark et al., 2007; Bhardwaja et al., 2012; Abrogoua et al., 2016; Lopes et al., 2017; Tang et al., 2018), and the use of a convenience sampling method (Lopes et al., 2017; Karuniawati et al., 2019).

## Discussion

### Main findings

The number of studies evaluating clinical pharmacy services in TB management is relatively scarce. Of note, none were randomized controlled trials, i.e., most were prospective studies with a non-randomized controlled design. In providing clinical pharmacy services, adherence measuring tools, medical records, patient interviews, and laboratory tests/therapeutic drug monitoring were frequently used as the clinical data source. Medication adherence and safety were the two intervention foci mostly provided in clinical pharmacy services for TB, followed by patient/caregiver educational needs/beliefs and medication/therapy effectiveness. Commonly conducted intervention activities were drug information/patient counseling, adverse drug reaction monitoring, drug use evaluation, and adherence monitoring. Educational materials/leaflets/written action plans, patient data collection forms, validated adherence questionnaires, and medical records were typically used to support the intervention activities.

The measured clinical pharmacy service outcomes were commonly process outcomes, i.e., not using the WHO outcome classification, making it wide-ranging and complex to make quantitative interpretations and comparisons between studies and outcomes. Only two studies used the WHO outcome classification. Most of the outcomes showed an improvement.

Of the 10 studies included, the risk of bias per domain was mainly low due to the limitations to the non-randomized study design. Therefore, despite the promising potential, convincing evidence for pharmacy services in TB management is still lacking.

### Interpretation

Our search strategy retrieved relevant studies of pulmonary TB conducted primarily in high-burden countries; however, studies focusing on clinical pharmacy services for TB in other countries were limited. We found this in line with the trend of increasing TB research in high-burden countries with more context-specific research to meet its own national strategic plan goals toward TB elimination, where fundamental and epidemiology research is mainly conducted (Cazabon et al., 2017; World Health Organization, 2017; Nafade et al., 2018; Morishita et al., 2020), making studies on clinical pharmacy services in TB relatively uncommon. A review also highlighted this by stating that clinical pharmacy services primarily target specific medical conditions, but TB was not mentioned (Rotta et al., 2017).

In the studies we reviewed, clinical pharmacy services were given as a supplementary service, focusing on its utilization to enhance an existing TB care program, supported by multiple relevant patient-related data sources to provide the service appropriately (World Health Organization, 2017; World Health Organization, 2019c; Mahmoud, 2019). This practical type of study falls into an operational research category to maximize the effectiveness and efficiency of interventions that can contribute to achieving the desired TB outcome (Kumar et al., 2020; Morishita et al., 2020). However, the absence of sound randomized studies of clinical pharmacy services as part of existing TB care indicated that accurate assessments of the effect of clinical pharmacy services within TB care are difficult.

Most clinical pharmacy service studies did not always follow the consistent characterization of their components, which means that the content or terminology describing the detailed activities of the services tends to vary across different studies (Bonetti et al., 2020). However, as a part of broader multi-disciplinary services, pharmacists’ actions through clinical pharmacy services have a noticeable benefit, for example, in curbing microbial resistance (Padayatchi et al., 2016; Kunoor et al., 2021), especially in TB care. Using the DEPICT (Correr et al., 2013), we characterized the components of clinical pharmacy services and their outcomes in TB care. The two most common interventions in our review were focused on enhancing medication adherence and safety.

Medication adherence interventions, such as patient education, counseling, patient support (material or psychological), patient scheduling, and digital technology, improve TB treatment outcomes (Alipanah et al., 2018). Drug information or patient counseling was the most frequently conducted activity within clinical pharmacy services to influence and alter patients’ knowledge of TB (Venkatapraveen et al., 2012; Thomas et al., 2018; Karuniawati et al., 2019; Narayana et al., 2020), and we found that this knowledge was improving. The improvement was in agreement with a previous study showing that structured counseling improved patient/caregiver educational needs/beliefs on TB care-related knowledge (Sajjad et al., 2020). However, sometimes it was hindered by the different perceptions of information adequacy and reception between patients and health services (Moodley et al., 2020). In addition, comparing the improvement between studies is challenging due to different settings and assessment frameworks. Improved knowledge does not warrant better TB care outcomes. Nevertheless, it could be a means to build better communication with patients and can lead to medication adherence.

Regarding medication safety interventions, most of the studies we reviewed focused on resolving pharmaceutical care needs/issues raised during treatment (Clark et al., 2007; Lopes et al., 2017; Tang et al., 2018). With 66%–87% of issues resolved, this is comparable to a study mentioning that 74.1% of pharmaceutical care needs/issues were completely solved by pharmacist interventions (Garin et al., 2021). Only one study identified the prevalence of adverse drug reactions, and with the result of 24.5% (Abrogoua et al., 2016), it is within the range of 8%–85%, as identified by Singh et al. (2015). Our review showed that medication safety issues were well-handled by the pharmacist, as also highlighted by Phansalkar et al. on the prominent contribution of pharmacists to medication safety (Phansalkar et al., 2007).

Regarding TB outcomes of the interventions identified, the WHO outcome classification (Tang et al., 2018; Tanvejsilp et al., 2018) and sputum conversion (Venkatapraveen et al., 2012; Tang et al., 2018) were generally improved through drug information or patient counseling activities. This was in agreement with studies stating that health education improves TB prognosis (Westerlund et al., 2015) and treatment completion (Alipanah et al., 2018), leading to a reduction in the treatment default rate (Müller et al., 2018).

Other than the WHO outcomes, adherence-related outcomes were among the most used. Measures for adherence included isoniazid metabolite presence (Clark et al., 2007), attendance (Clark et al., 2007; Tang et al., 2018), adherence level (Karuniawati et al., 2019), compliance (Karuniawati et al., 2019), medication counting (Clark et al., 2007; Tang et al., 2018), and the Morisky Adherence Questionnaire Scale (Bhardwaja et al., 2012; Thomas et al., 2018). Although the methods were diverse and adherence is only seen as an intermediate outcome, a study providing drug information and patient counseling activities did improve treatment outcomes (Alipanah et al., 2018), disregarding the method of measuring adherence (Valencia et al., 2017). HRQoL outcomes were also used (Tanvejsilp et al., 2018), yet conflicting results were observed, as also noted by a study about their negative impact on the various domains of the quality of life during long-term TB care (Salehitali et al., 2019). We found that quality of life improvements through clinical pharmacy services are still scarce; only one very recent study provided RCT evidence (Khan et al., 2023), making it difficult to draw any general conclusions on the impact of clinical pharmacy services on patients’ quality of life.

Most studies in this review were adequately carried out despite their non-randomized design and could provide preliminary evidence on the benefit of clinical pharmacy service interventions, especially single interventions. Still, due to the nature of observational studies, prone to undetected confounding and bias, their results are not comparable to a well-designed randomized trial.

Pharmacy services generally consist of composite interventions, making it challenging to directly meta-analyze their overall effect (Teoh et al., 2019; Bonetti et al., 2020). The association between drug information and patient counseling with TB-related knowledge, medication adherence, medication effectiveness, and quality of life in improving TB care-related outcomes is complex. We anticipate that improving knowledge through structured education will lead to better adherence, improved medication effectiveness, and achieving TB care treatment goals, which will benefit the quality of life of patients with TB in the long run.

### Strengths and limitations

The strength of this review is the use of a standardized framework for identifying and assessing clinical pharmacy services as single or composite intervention activities in TB management, resulting in a consistent reporting of clinical pharmacy service component identification. A limitation is focusing on English language publications only and, therefore, retrieving studies from a limited number of countries, potentially missing relevant studies in high-burden countries that use their national language. In addition, the analysis was restricted to the effect of clinical pharmacy services in patients with drug-susceptible TB only. Even while this resulted in more focus, due to the heterogeneity of the outcomes identified, a quantitative analysis of the effectiveness of clinical pharmacy services was not possible.

## Recommendations

Identification and prioritization of the provision for clinical pharmacy services for TB management need to be established by increasing operational research activities and, at the same time, providing high-quality effectiveness evidence of clinical pharmacy services in TB management through randomized controlled trials, aside from process outcomes, which directly measure clinical pharmacy service process accomplishments; WHO outcomes should be included as the main outcome of interest in evaluating the clinical pharmacy service impact. The integration of clinical pharmacy services in our daily routine for TB care management is necessary to support patient-centered care practices and to improve TB care management outcomes.

## Conclusion

Although clinical pharmacy services as single or composite interventions potentially improve TB outcomes, its evidence is still inconsistent and limited due to the lack of randomized controlled studies using the WHO outcome classification. Furthermore, well-designed RCTs are required for supporting larger-scale implementations.

## Registration and protocol

In accordance with the guidelines, our systematic review protocol was registered with the PROSPERO on 15 August 2020 and was last updated on 18 January 2021 (registration number CRD42020199028).

## Data Availability

The original contributions presented in the study are included in the article/Supplementary Material; further inquiries can be directed to the corresponding author.
