# Supplementary material for: Clinical pharmacy services for tuberculosis management: a systematic review
Source: Front Pharmacol. 2023 Jul 7;14:1186905. doi: 10.3389/fphar.2023.1186905 (PMC10360183; doi:10.3389/fphar.2023.1186905)
Supplement: Supplementary file 1 [file DataSheet1.docx]

Clinical Pharmacy Services for Tuberculosis Management:

A Systematic Review

*D. Iskandar, F.D.A. Suryanegara, J.F.M. van Boven, M.J. Postma*

# APPENDIX

Contents

[APPENDIX 1](#_Toc116472440)

[1. Search strategy 4](#_Toc116472441)

[1.1. Pubmed 4](#_Toc116472442)

[1.2. Embase 4](#_Toc116472443)

[1.3. Cochrane 4](#_Toc116472444)

[1.4. Scopus 4](#_Toc116472445)

[1.5. Web of science 4](#_Toc116472446)

[2. Article screening 5](#_Toc116472447)

[3. Descriptive Elements of Pharmacist Intervention Characterization 11](#_Toc116472448)

[4. Clinical pharmacy services 13](#_Toc116472449)

[4.1. Clinical data sources 15](#_Toc116472450)

[4.2. Intervention focus 15](#_Toc116472451)

[4.3. Intervention activities 16](#_Toc116472452)

[4.4. Supporting material 16](#_Toc116472453)

[4.5. Other outcomes in a single group measurement 18](#_Toc116472454)

[REFERENCES 22](#_Toc116472455)

**Tables**

[Table 1. Article screening criteria 5](#_Toc116472456)

[Table 2. Taxonomy for identifying study design classifications(1) 10](#_Toc116472457)

[Table 3. Descriptive Elements of Pharmacist Intervention Characterization Tool (DEPICT)(2, 3) 11](#_Toc116472458)

[Table 4. Clinical pharmacy services components for TB care management 13](#_Toc116472459)

[Table 5. Intervention activity based on intervention focus 16](#_Toc116472460)

[Table 6. Classification of clinical pharmacy services intervention outcomes for TB care management 17](#_Toc116472461)

[Table 7. Other outcomes of clinical pharmacy services for TB care management in a single group measurement 18](#_Toc116472462)

**Figures**

[Figure 1. Article screening flowchart 8](#_Toc116472463)

[Figure 2. Bibliometric analysis 9](#_Toc116472464)

[Figure 3. Clinical data sources in clinical pharmacy services for TB care management 15](#_Toc116472465)

[Figure 4. Intervention focuses in clinical pharmacy services for TB care management 15](#_Toc116472466)

[Figure 5. Supporting materials in clinical pharmacy services for TB care management 16](#_Toc116472467)

# Search strategy

## Pubmed

("Tuberculosis"[MeSH Terms] OR "Tuberculosis"[Title/Abstract] OR "tb"[Title/Abstract]) AND ("Pharmacy"[MeSH Terms] OR "evidence-based pharmacy practice"[MeSH Terms] OR "Pharmacy Research"[MeSH Terms] OR "pharmacy service, hospital"[MeSH Terms] OR "Pharmaceutical Services"[MeSH Terms] OR "Pharmacy and Therapeutics Committee"[MeSH Terms] OR "Pharmacists"[MeSH Terms] OR "pharmaci*"[Title/Abstract] OR "pharmacy*"[Title/Abstract] OR "pharmaceu*"[Title/Abstract])

## Embase

('tuberculosis' OR 'tuberculosis':ti,ab OR 'tb':ti,ab) AND (('pharmacy'/exp OR pharmacy) AND ('discipline'/exp OR discipline) OR 'evidence-based pharmacy' OR 'pharmacy research' OR 'hospital pharmacy service' OR 'pharmacy (shop)' OR (pharmacy AND therapeutics AND committee) OR 'pharmacist' OR 'pharmaceutical care')

## Cochrane

(MeSH descriptor: [Tuberculosis] explode all trees OR tuberculosis:ti,ab,kw OR tb:ti,ab,kw) AND (MeSH descriptor: [Pharmacy] explode all trees OR MeSH descriptor: [Pharmaceutical Services] explode all trees OR MeSH descriptor: [Pharmacy Service, Hospital] explode all trees OR MeSH descriptor: [Pharmacists] explode all trees OR pharmaci*:ti,ab,kw OR pharmacy*:ti,ab,kw OR pharmaceu*:ti,ab,kw)

## Scopus

((TITLE-ABS-KEY (tuberculosis) OR TITLE-ABS-KEY (tb)) AND DOCTYPE (ar)) AND (((TITLE-ABS-KEY (clinical)) AND ((TITLE-ABS-KEY (pharmacy) OR TITLE-ABSKEY (pharmacist*))) AND DOCTYPE (ar)) OR ((TITLE-ABS-KEY (hospital)) AND ((TITLEABS-KEY (pharmacy) OR TITLE-ABS-KEY (pharmacist*))) AND DOCTYPE (ar)) OR (((TITLE-ABS-KEY (pharmacy) OR TITLE-ABS-KEY (pharmaceutical) OR TITLE-ABSKEY (pharmacist*))) AND ((TITLE-ABS-KEY (service*) OR TITLE-ABS-KEY (care))) AND DOCTYPE (ar)))

## Web of science

(((((AB=(pharmaceu*)) AND LA=(English)) AND DT=(Article)) OR (((AB=(pharmacy*)) AND LA=(English)) AND DT=(Article)) OR (((AB=(pharmaci*)) AND LA=(English)) AND DT=(Article)) OR (((TI=(pharmaceu*)) AND LA=(English)) AND DT=(Article)) OR (((TI=(pharmacy*)) AND LA=(English)) AND DT=(Article)) OR (((TI=(pharmaci*)) AND LA=(English)) AND DT=(Article))) OR ((((TS=(hospital AND pharmacist*)) AND LA=(English)) AND DT=(Article)) OR (((TS=(hospital pharmacy)) AND LA=(English)) AND DT=(Article)) OR (((TS=(clinical AND pharmacist*)) AND LA=(English)) AND DT=(Article)) OR (((TS=(clinical pharmacy)) AND LA=(English)) AND DT=(Article)) OR (((TS=(pharmacy AND service*)) AND LA=(English)) AND DT=(Article)) OR (((TS=(pharmacy care)) AND LA=(English)) AND DT=(Article)) OR (((TS=(pharmacist AND service*)) AND LA=(English)) AND DT=(Article)) OR (((TS=(pharmacist care)) AND LA=(English)) AND DT=(Article)) OR (((TS=(pharmaceutical AND service*)) AND LA=(English)) AND DT=(Article)) OR (((TS=(pharmaceutical care)) AND LA=(English)) AND DT=(Article)))) AND ((((TS=(tuberculosis)) AND LA=(English)) AND DT=(Article)) OR (((TI=(tuberculosis OR tb)) AND LA=(English)) AND DT=(Article)))

# Article screening

Table S1. Article screening criteria

| No | Criteria | Requirements | Exclusion reason | Details | Code |
| --- | --- | --- | --- | --- | --- |
| 1 | Language | English | Foreign language | Non-English language | 1 |
| 2 | Type of publication | Original research, full text, non-review, non-editorial, non-commentary, non-letter | Wrong publication type | Non-original article, unrelated to TB/clinical pharmacy service in TB | 2 |
|  |  |  | Wrong publication type/background article | Non-original article, related to TB/clinical pharmacy service in TB | 2 |
| 3 | Type of study | Related to TB | Wrong study design | Non-tuberculosis study | 3 |
|  |  | Case report | Wrong study design | Non-clinical study | 4 |
|  |  | Case series |  |  |  |
|  |  | Survey (cross-sectional) |  |  |  |
|  |  | Qualitative |  |  |  |
|  |  | Cross-sectional (analytic) |  |  |  |
|  |  | Case-control |  |  |  |
|  |  | Cohort |  |  |  |
|  |  | Randomized controlled trial (parallel) |  |  |  |
|  |  | Randomized controlled trial (crossover) |  |  |  |
|  |  | Randomized controlled trial (pragmatic) |  |  |  |
|  |  | Quasi-experimental |  |  |  |
|  |  | Non-randomized controlled trial |  |  |  |
|  |  | Unclear |  |  |  |
|  |  | Other |  |  |  |
| 4 | Type of intervention | Prescription screening and dispensing | Wrong study design | Non-clinical pharmacy study | 9 |
|  |  | Admission Drug Histories |  |  |  |
|  |  | Drug Use Review |  |  |  |
|  |  | Drug Reconciliation |  |  |  |
|  |  | Drug Information Service |  |  |  |
|  |  | Counseling/education |  |  |  |
|  |  | Visit/Round |  |  |  |
|  |  | Drug Therapy Monitoring |  |  |  |
|  |  | Adverse Drug Reaction Monitoring |  |  |  |
|  |  | Drug Use Evaluation |  |  |  |
|  |  | Sterile Dosage form Dispensing |  |  |  |
|  |  | Drug Blood-level Monitoring |  |  |  |
|  |  | Drug Protocol Management |  |  |  |
|  |  | Collaborative Practice |  |  |  |
|  |  | Immunization |  |  |  |
|  |  | Primary Care |  |  |  |
| 5 | Type of subject | Human | Wrong study design/wrong population | Non-human study | 5 |
|  |  | Diagnosed with pulmonary TB | Wrong population | Non-pulmonary TB | 7 |
|  |  |  |  | Latent TB |  |
|  |  |  |  | TB-HIV | 8 |
|  |  |  |  | TB-comorbidity |  |
|  |  | 18 Years of age | Wrong population | Not adult | 6 |
| 6 | Type of drug | Anti-tuberculosis | Wrong study design/wrong drug | Non-clinical pharmacy study, non-tuberculosis medication | 10 |
| 7 | Type of outcome | Primary | Wrong study design/wrong outcome/background article wrong study design/background article | Non-clinical pharmacy study, related to TB, non-interested outcome | 11 |
|  |  | Cured |  |  |  |
|  |  | Treatment completed |  |  |  |
|  |  | Treatment failed |  |  |  |
|  |  | Died |  |  |  |
|  |  | Lost to follow up |  |  |  |
|  |  | Not evaluated |  |  |  |
|  |  | Treatment success |  |  |  |
|  |  |  |  |  |  |
|  |  | Secondary |  |  |  |
|  |  | Bioavailability/bioequivalence |  | Non-clinical pharmacy study, non-interested outcome | 12 |
|  |  | Chemical/biological analysis |  |  |  |
|  |  | Metabolism |  |  |  |
|  |  | Prescribing pattern |  |  |  |
|  |  | Compliance/adherence |  |  |  |
|  |  | Knowledge, attitude, practice |  |  |  |
|  |  | Pharmacology/activity |  |  |  |
|  |  | Risk factors |  |  |  |
|  |  | Safety, efficacy |  |  |  |
|  |  | Economic evaluation |  |  |  |


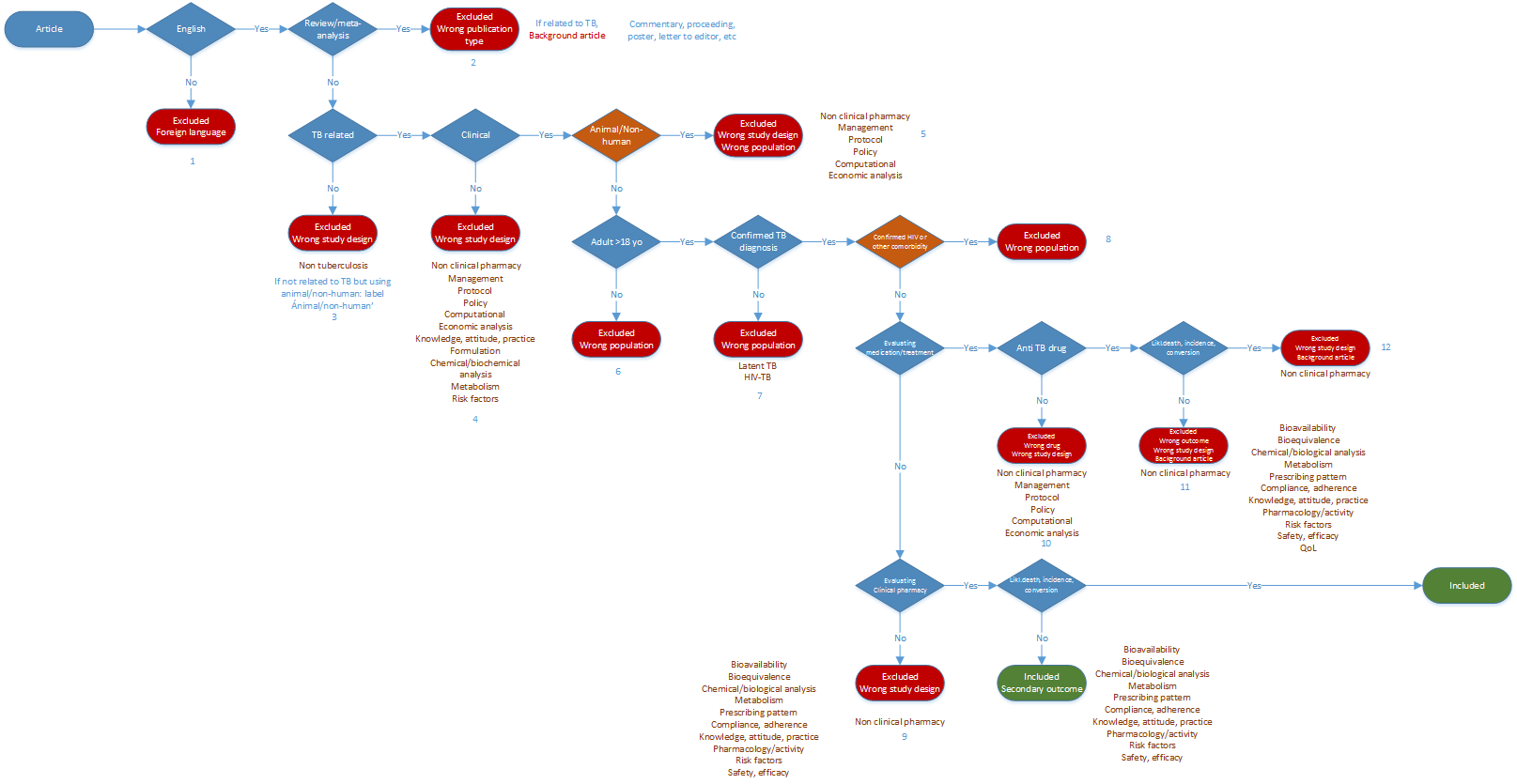


Figure S1. Article screening flowchart


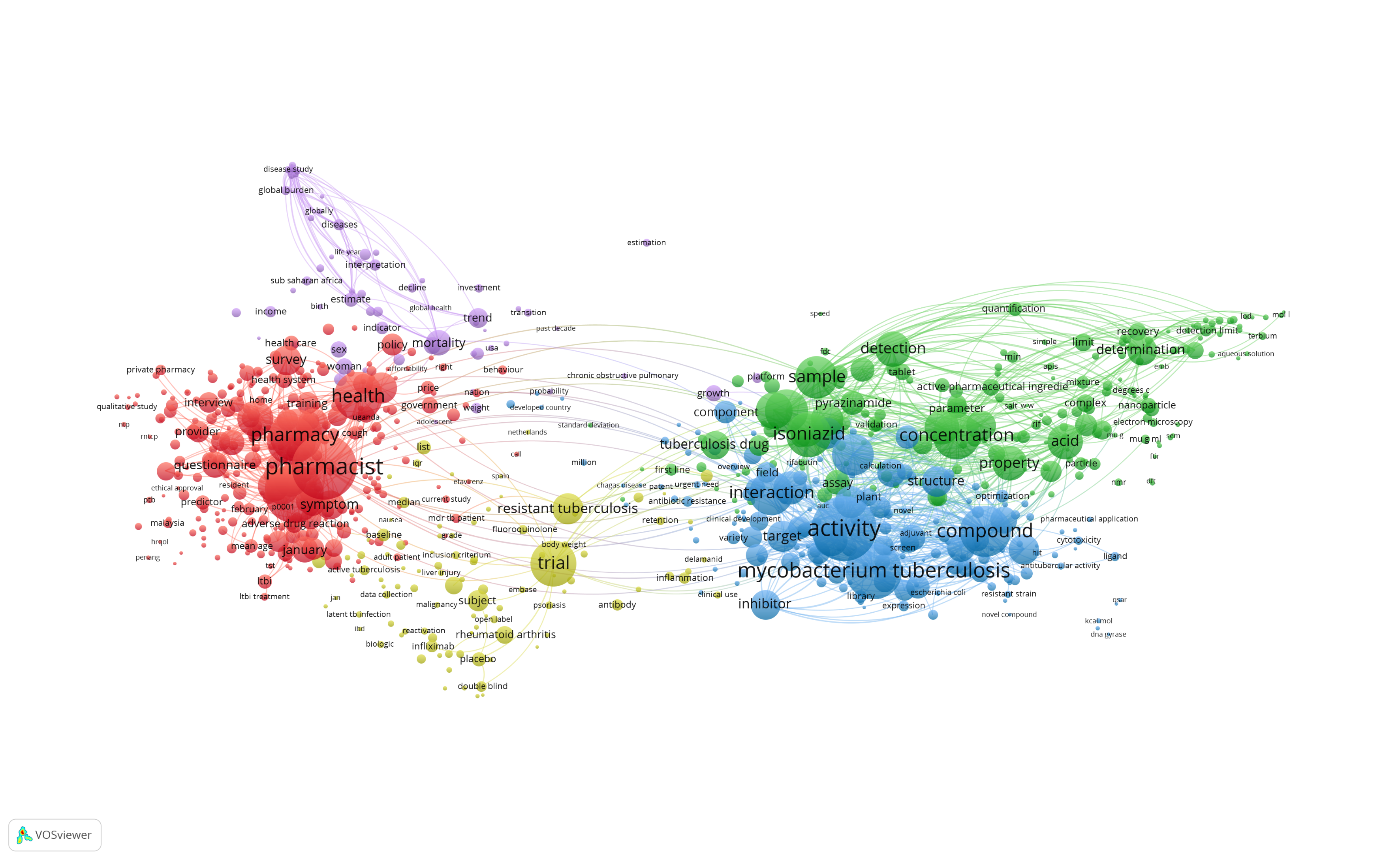


Figure S2. Bibliometric analysis

Table S2. Taxonomy for identifying study design classifications(1)

| **#** | **Question** | **If answer is yes:** | **If answer is no:** |
| --- | --- | --- | --- |
| 1 | Was there a comparison to assess the effect/association of an intervention/exposure and an outcome? (This could be between ≥2 groups or within the same individual or group(s) comparing pre- and post intervention measures) | Skip to #2 | Non-comparative study (e.g. case report or case series |
| 2 | Was information on the intervention/exposure and outcome gathered by the investigators concurrently (e.g. by survey)? | Cross-sectional study | Skip to #3 |
| 3 | Were participants (or clusters) allocated to ≥2 study groups by actions of the researchers (i.e., decisions or systems applied by the researchers, such as randomization or quasi-randomized methods)? | Skip to #4 | Skip to #5 |
| 4 | Were interventions/exposures assigned randomly? | Randomized controlled trial | Non-randomized controlled trial (E.g. quasi-randomized controlled trial) |
| 5 | Did all the participants receive the same intervention/exposure? | Skip to #9 | Skip to #6 |
| 6 | Was there within group comparisons of participants over time (i.e., before/after or pre/post measurements)? | Skip to #10 | Skip to #7 |
| 7 | Were groups defined by the intervention/exposure? | Skip to #11 | Skip to #8 |
| 8 | Were intervention/exposure data registered prior to the outcome (e.g. from an existing longitudinal study)? | Nested case control study | Case control study |
| 9 | Were there ≥3 measurements before & ≥3 measurements after the intervention/exposure? | Interrupted time series (without comparison group | Before-after study |
| 10 | Were there ≥3 measurements before & ≥3 measurements after the intervention/exposure? | Interrupted time series (with comparison group) | Controlled before-after study |
| 11 | Were groups concurrent? | Skip to #12 | Non-concurrent cohort study |
| 12 | Were both intervention/exposure and outcome assessed prospectively? | Prospective cohort study | Retrospective cohort study |

# Descriptive Elements of Pharmacist Intervention Characterization

Table S3. Descriptive Elements of Pharmacist Intervention Characterization Tool (DEPICT)(2, 3)

# Clinical pharmacy services

Table S4. Clinical pharmacy services components for TB care management

| Author, year | Clinical data source | Intervention focus | Intervention activity | Supporting material |
| --- | --- | --- | --- | --- |
| Abrogoua,  2016 (4)** | - Direct contact with HCP - DRP and PI dashboard - Medical records | - Drug selection (Rx, OTC or other) - Medication safety | - Change or suggestion for change in therapy/lab test-Drug use evaluation - Monitoring result report-Adverse drug reaction monitoring - Prescription screening | - Guidelines / Clinical protocols / Evidence chart - Medical records - Medication list / Medication schedule / Medication report |
| Bhardwaja,  2012 (5)* | - - Adherence measuring tools   - Medical records   - Patient interview (not including assessment procedures or tests) | Medication adherence | Drug information or patient counseling | - - Educational materials / Leaflets / Written action plan   - Medication list / Medication schedule / Medication report   - Morisky Medication Adherence Questionnaire   - Patient data collection form |
| Clark, 2007 (6)** | - - Adherence measuring tools   - Direct contact with HCP   - Laboratory tests/therapeutic drug monitoring   - Medical records   - Patient interview (not including assessment procedures or tests) | - - Medication adherence   - Medication safety | - - Drug information or patient counseling   - Monitoring result report-Adverse drug reaction monitoring | - - Educational materials / Leaflets / Written action plan |
| Karuniawati, 2019 (7)* | Adherence measuring tools | Medication adherence | Drug information or patient counseling | - Educational materials / Leaflets / Written action plan - Validated adherence questionnaire |
| Lopes, 2017 (8)* | - ADR detection questionnaire - Patient interview (not including assessment procedures or tests) - Pharmacotherapy workup questionnaire | Medication safety | Monitoring result report-Adverse drug reaction monitoring | - Pharmacotherapy workup questionnaire - Standardized questionnaire for the detection of adverse reaction |
| Narayana,  2020 (9)* | - Adherence measuring tools - knowledge questionnaire | Patient / Caregiver educational needs / Beliefs | Drug information or patient counseling | - Educational materials / Leaflets / Written action plan - Validated adherence questionnaire |
| Tang, 2018 (10)** | - Adherence measuring tools - Direct contact with HCP - Laboratory tests/therapeutic drug monitoring - Medical records | - Medication / Therapy effectiveness - Medication adherence - Medication safety | - Change or suggestion for change in therapy/lab test-Drug use evaluation - Drug information or patient counseling - Monitoring result report-Adverse drug reaction monitoring - Monitoring result report-Drug therapy monitoring - Update of patient's medication list | Educational materials / Leaflets / Written action plan |
| Tanvejsilp,  2018 (11)** | - Medical records - Patient interview (not including assessment procedures or tests) - TB registration records | - HRQoL - Medication / Therapy effectiveness | - Adherence monitoring - Drug information or patient counseling - Monitoring result report-Adverse drug reaction monitoring | - EQ5D3L - Medical records - Patient data collection form - TB registration records |
| Thomas,  2018 (12)* | - Adherence measuring tools - Laboratory tests/therapeutic drug monitoring - Medical records - Patient interview (not including assessment procedures or tests) | - Medication adherence - Patient / Caregiver educational needs / Beliefs | Drug information or patient counseling | - Auxiliary labels / Pictorial instructions / Written reminders - Educational materials / Leaflets / Written action plan - Knowledge assessment - Morisky Medication Adherence Questionnaire - Patient data collection form |
| Venkatapraveen, 2012 (13)* | - Adherence measuring tools - knowledge questionnaire - Laboratory tests/therapeutic drug monitoring | - Medication / Therapy effectiveness - Patient / Caregiver educational needs / Beliefs | Drug information or patient counseling | - Educational materials / Leaflets / Written action plan - Knowledge assessment - Patient data collection form - Validated adherence questionnaire |

**Single intervention activity*

***Composite intervention activities*

## Clinical data sources

Figure S3. Clinical data sources in clinical pharmacy services for TB care management

## Intervention focus

Figure S4. Intervention focuses in clinical pharmacy services for TB care management

## Intervention activities

Table S5. Intervention activity based on intervention focus

| Intervention focus | Intervention activity |
| --- | --- |
| Drug selection | Drug use evaluation |
|  | Prescription screening |
| HRQoL | Adherence monitoring |
|  | Drug information/patient counseling |
|  | Adverse drug reaction monitoring |
| Medication/therapy effectiveness | Adherence monitoring |
|  | Drug use evaluation |
|  | Adverse drug reaction monitoring |
|  | Drug therapy monitoring |
| Medication adherence | Drug use evaluation |
|  | Drug information/patient counseling |
|  | Update of patient's medication list |
| Medication safety | Adverse drug reaction monitoring |
| Patient/caregiver educational needs/beliefs | Drug information/patient counseling |

## Supporting material

Figure S5. Supporting materials in clinical pharmacy services for TB care management

Table S6. Classification of clinical pharmacy services intervention outcomes for TB care management

| Outcome | | Number of studies (%) |
| --- | --- | --- |
| WHO outcomes | Treatment success | 1 (10) |
|  | Treatment completion | 2 (20) |
|  | Cure | 2 (20) |
|  | Default | 2 (20) |
|  | Death | 2 (20) |
|  | Not completed | 1 (10) |
|  | Transfer out | 1 (10) |
|  | Failure | 1 (10) |
| Other outcomes | Medication counting | 2 (20) |
|  | Pharmaceutical care needs | 2 (20) |
|  | Morisky adherence questionnaire scale | 2 (20) |
|  | Attendance | 2 (20) |
|  | Isoniazid metabolites | 2 (20) |
|  | Knowledge | 2 (20) |
|  | Pharmaceutical care impact index | 1 (10) |
|  | Sputum conversion time (months) | 1 (10) |
|  | Pharmaceutical intervention acceptance rate | 1 (10) |
|  | Adherence barrier: psychological | 1 (10) |
|  | Adherence barrier: personal | 1 (10) |
|  | VAS | 1 (10) |
|  | Compliance | 1 (10) |
|  | Adherence barrier: knowledge | 1 (10) |
|  | Sputum conversion | 1 (10) |
|  | Medication adherence | 1 (10) |
|  | EQ5D3L score | 1 (10) |
|  | Adherence level | 1 (10) |
|  | Knowledge and adherence score | 1 (10) |

## Other outcomes in a single group measurement

Table S7. Other outcomes of clinical pharmacy services for TB care management in a single group measurement

| **Outcome** | | | |  | **Lopes, 2017** (8) |  | **Clark, 2007** (6) |  | **Tang, 2018 (10)** |  | **Abrogoua, 2016** (4) |  | **Narayana, 2020** (9) |  | **Thomas,**  **2018** (12) |  | **Bhardwaja, 2012** (5) |
| --- | --- | --- | --- | --- | --- | --- | --- | --- | --- | --- | --- | --- | --- | --- | --- | --- | --- |
|  |  |  |  |  | Pharmaceutical care |  | Pharmaceutical interventions |  | Pharmaceutical interventions |  | Pharmaceutical interventions |  | Education |  | Education |  | Education |
| **Measured at the end of the intensive and continuation phase** | | | | | | | |  |  |  |  |  |  |  |  |  |  |
|  | Pharmaceutical care impact index | | |  | 0.66±0.38 |  | - |  | - |  | - |  | - |  | - |  | - |
|  | Pharmaceutical care needs | | |  | - |  | 75.00%* |  | 87.72%* |  | 24.5% |  | - |  | - |  | - |
|  | Pharmaceutical intervention acceptance rate | | |  | - |  | - |  | - |  | 100% |  | - |  | - |  | - |
|  |  |  |  |  |  |  |  |  |  |  |  |  |  |  |  |  |  |
| **Measured at follow-up time interval within the intensive and continuation phase** | | | | | | | | | |  |  |  |  |  |  |  |  |
|  | Medication adherence | | |  |  |  |  |  |  |  |  |  |  |  |  |  |  |
|  |  | Baseline | |  | - |  | - |  | - |  | - |  | 87.41±6.23% |  | - |  | - |
|  |  | 1st follow-up | |  | - |  | - |  | - |  | - |  | 92.83±3.77% |  | - |  | - |
|  |  | 2nd follow-up | |  | - |  | - |  | - |  | - |  | 98.88±1.34% |  | - |  | - |
|  |  | Sig. | |  | - |  | - |  | - |  | - |  | < 0.0001 |  | - |  | - |
|  | Knowledge | | |  |  |  |  |  |  |  |  |  |  |  |  |  |  |
|  |  | Baseline | |  | - |  | - |  | - |  | - |  | 5.22±2.37 |  | 21±3.9 |  | - |
|  |  | 1st follow-up | |  | - |  | - |  | - |  | - |  | 10.32±2.53 |  | 24.74±3.36 |  | - |
|  |  | 2nd follow-up | |  | - |  | - |  | - |  | - |  | 12.43±2.59 |  | 28.34±3 |  | - |
|  |  | Final | |  | - |  | - |  | - |  | - |  | - |  | 31.72±3.049 |  | - |
|  |  | Sig. | |  | - |  | - |  | - |  | - |  | - |  | 0.05 |  | - |
|  |  |  | 1st follow-up to baseline |  | - |  | - |  | - |  | - |  | < 0.0001 |  | - |  | - |
|  |  |  | 2nd follow-up to 1st follow-up |  | - |  | - |  | - |  | - |  | < 0.0001 |  | - |  | - |
|  |  |  | 2nd follow-up to baseline |  | - |  | - |  | - |  | - |  | < 0.0001 |  | - |  | - |
|  | Adherence barrier: knowledge | | |  |  |  |  |  |  |  |  |  |  |  |  |  |  |
|  |  | Baseline | |  | - |  | - |  | - |  | - |  | - |  | 11.22±3.2 |  | - |
|  |  | 1st follow-up | |  | - |  | - |  | - |  | - |  | - |  | 13.38±2.83 |  | - |
|  |  | 2nd follow-up | |  | - |  | - |  | - |  | - |  | - |  | 15.29±2.5 |  | - |
|  |  | Final | |  | - |  | - |  | - |  | - |  | - |  | 19.29±1.37 |  | - |
|  | Adherence barrier: personal | | |  |  |  |  |  |  |  |  |  |  |  |  |  |  |
|  |  | Baseline | |  | - |  | - |  | - |  | - |  | - |  | 4.2±1.121 |  | - |
|  |  | 1st follow-up | |  | - |  | - |  | - |  | - |  | - |  | 4.71±0.996 |  | - |
|  |  | 2nd follow-up | |  | - |  | - |  | - |  | - |  | - |  | 5.63±0.911 |  | - |
|  |  | Final | |  | - |  | - |  | - |  | - |  | - |  | 6.63±0.601 |  | - |
|  | Adherence barrier: psychological | | |  |  |  |  |  |  |  |  |  |  |  |  |  |  |
|  |  | Baseline | |  | - |  | - |  | - |  | - |  | - |  | 5.63±1.58 |  | - |
|  |  | 1st follow-up | |  | - |  | - |  | - |  | - |  | - |  | 6.71±1.221 |  | - |
|  |  | 2nd follow-up | |  | - |  | - |  | - |  | - |  | - |  | 7.4±0.997 |  | - |
|  |  | Final | |  | - |  | - |  | - |  | - |  | - |  | 8.26±0.776 |  | - |
|  | Morisky adherence questionnaire scale | | |  |  |  |  |  |  |  |  |  |  |  |  |  |  |
|  |  | Baseline | |  | - |  | - |  | - |  | - |  | - |  | 3.615±1.674 |  | 2.25 |
|  |  | 1st follow-up | |  | - |  | - |  | - |  | - |  | - |  | 2.723±1.625 |  | 3.97 |
|  |  | 2nd follow-up | |  | - |  | - |  | - |  | - |  | - |  | 1.4±1.072 |  | 4.69 |
|  |  | Final | |  | - |  | - |  | - |  | - |  | - |  | 0.123±0.45 |  | - |
|  |  | Sig. | |  | - |  | - |  | - |  | - |  | - |  | < 0.05 |  | - |
|  |  |  | 1st follow-up to baseline |  | - |  | - |  | - |  | - |  | - |  | - |  | < 0.0001 |
|  |  |  | 2nd follow-up to baseline |  | - |  | - |  | - |  | - |  | - |  | - |  | < 0.0001 |

**Calculated from resolved cases divided by total cases requiring pharmaceutical care*

ADR: Adverse Drug Reaction

PI: Pharmacist Intervention

TB: Tuberculosis

HCP: Health Care Professional

HRQoL: Health-Related Quality of Life

VAS: Visual Analogue Score

DRP: Drug-Related Problem

WHO: World Health Organization

REFERENCES

1. Viswanathan M, Berkman ND, Dryden DM, Hartling L. *Assessing Risk of Bias and Confounding in Observational Studies of Interventions or Exposures: Further Development of the RTI Item Bank*. Rockville (MD) (2013). 49 p.

2. *Descriptive Elements of Pharmacist Intervention Characterization Tool: DEPICT Manual of instructions*. Available from: http://depictproject.org/downloads_files/Depict_Version_2_manual.pdf

3. *Descriptive Elements of Pharmacist Intervention Characterization Tool: DEPICT 2*. Available from: http://depictproject.org/downloads_files/Depict_tool_version2.pdf

4. Abrogoua DP, Kamenan BA, Ahui BJ, Doffou E. Pharmaceutical interventions in the management of tuberculosis in a pneumophtisiology department, Ivory Coast. *Therapeutics and clinical risk management* (2016) **12**:1749–56. doi:10.2147/TCRM.S118442

5. Bhardwaja A., Kumar R., Dabas V., Alam N. Assessment and enhancing adherence to treatment regimen in tuberculosis out patients. *Int. J. Pharmcy Pharm. Sci. International Journal of Pharmacy and Pharmaceutical Sciences* (2012) **4**:517–22.

6. Clark PM, Karagoz T, Apikoglu-Rabus S, Izzettin FV. Effect of pharmacist-led patient education on adherence to tuberculosis treatment. *American journal of health-system pharmacy AJHP official journal of the American Society of Health-System Pharmacists* (2007) **64**:497–505. doi:10.2146/ajhp050543

7. Karuniawati H, Putra ON, Wikantyasning ER. Impact of pharmacist counseling and leaflet on the adherence of pulmonary tuberculosis patients in lungs hospital in Indonesia. *Indian J Tuberc* (2019) **66**:364–9. doi:10.1016/j.ijtb.2019.02.015

8. Lopes AR, Miranda SS de, Ceccato MD, Silveira MR, Resende NH de, Carvalho WS. Evaluation of the impact of pharmaceutical care for tuberculosis patients in a Secondary Referral Outpatient Clinic, Minas Gerais, Brazil. *Anais da Academia Brasileira de Ciencias* (2017) **89**:2911–9. doi:10.1590/0001-3765201720170301

9. Narayana G, Jyoshna K, Kishore M, Pradeepkumar B, Bogireddy S, Smg I, et al. Impact Of Pharmacist Counselling On Knowledge, And Medication Adherence In Tuberculosis Patients: A Quasi-Experimental Design. *Int J Pharma Bio Sci* (2020) **10**. doi:10.22376/ijpbs/lpr.2020.10.5.P6-10

10. Tang Z-Q, Jiang R-H, Xu H-B. Effectiveness of pharmaceutical care on treatment outcomes for patients with first-time pulmonary tuberculosis in China. *J Clin Pharm Ther* (2018) **43**:888–94. doi:10.1111/jcpt.12746

11. Tanvejsilp P, Loeb M, Dushoff J, Xie F. Out-of-Pocket Expenditures, Indirect Costs and Health-Related Quality of Life of Patients with Pulmonary Tuberculosis in Thailand. *PharmacoEconomics - open* (2018) **2**:281–96. doi:10.1007/s41669-017-0057-9

12. Thomas A, Joy J, Kurian A, V S. SOCIO-EPIDEMIOLOGICAL EVALUATION OF TUBERCULOSIS AND IMPACT OF PHARMACEUTICAL CARE ON MEDICATION ADHERENCE AMONG TUBERCULOSIS PATIENTS. *Asian J Pharm Clin Res* (2018) **11**:265. doi:10.22159/ajpcr.2018.v11i2.20503

13. Venkatapraveen A, Rampure MV, Patil N, Hinchageri SS, Lakshmi DP. Assessment of clinical pharmacist intervention to improve compliance and health care outcomes of tuberculosis patients. *Der Pharmacia Lettre* (2012) **4**:931–7.
